# Application of a New Engineered Strain of *Yarrowia lipolytica* for Effective Production of Calcium Ketoglutarate Dietary Supplements

**DOI:** 10.3390/ijms22147577

**Published:** 2021-07-15

**Authors:** Ludwika Tomaszewska-Hetman, Anita Rywińska, Zbigniew Lazar, Piotr Juszczyk, Magdalena Rakicka-Pustułka, Tomasz Janek, Marta Kuźmińska-Bajor, Waldemar Rymowicz

**Affiliations:** Department of Biotechnology and Food Microbiology, Wrocław University of Environmental and Life Sciences, Chełmońskiego Street 37, 51-630 Wrocław, Poland; anita.rywinska@upwr.edu.pl (A.R.); zbigniew.lazar@upwr.edu.pl (Z.L.); piotr.juszczyk@upwr.edu.pl (P.J.); magdalena.rakicka-pustulka@upwr.edu.pl (M.R.-P.); tomasz.janek@upwr.edu.pl (T.J.); marta.kuzminska-bajor@upwr.edu.pl (M.K.-B.); waldemar.rymowicz@upwr.edu.pl (W.R.)

**Keywords:** *Yarrowia lipolytica*, α-ketoglutaric acid (KGA), calcium ketoglutarate, dietary supplement, metabolic engineering, glycerol, rapeseed oil, bioreactor fed-batch culture

## Abstract

The present study aimed to develop a technology for the production of dietary supplements based on yeast biomass and α-ketoglutaric acid (KGA), produced by a new transformant of *Yarrowia lipolytica* with improved KGA biosynthesis ability, as well to verify the usefulness of the obtained products for food and feed purposes. Transformants of *Y. lipolytica* were constructed to overexpress genes encoding glycerol kinase, methylcitrate synthase and mitochondrial organic acid transporter. The strains were compared in terms of growth ability in glycerol- and oil-based media as well as their suitability for KGA biosynthesis in mixed glycerol–oil medium. The impact of different C:N:P ratios on KGA production by selected strain was also evaluated. Application of the strain that overexpressed all three genes in the culture with a C:N:P ratio of 87:5:1 allowed us to obtain 53.1 g/L of KGA with productivity of 0.35 g/Lh and yield of 0.53 g/g. Finally, the possibility of obtaining three different products with desired nutritional and health-beneficial characteristics was demonstrated: (1) calcium α-ketoglutarate (CaKGA) with purity of 89.9% obtained by precipitation of KGA with CaCO_3_, (2) yeast biomass with very good nutritional properties, (3) fixed biomass-CaKGA preparation containing 87.2 μg/g of kynurenic acid, which increases the health-promoting value of the product.

## 1. Introduction

α-Ketoglutarate (KGA) is an organic acid that is integral to the basic central metabolism of the cell life cycle. This intermediate of the Krebs cycle plays important role in the energy supply as well as metabolism of carbon and nitrogen [1]. In the cell, KGA can be rapidly converted to glutamic acid via transamination and then aminated to glutamine [2]. KGA is considered one of the crucial metabolic intermediates in protein metabolism, amino acid transport across membranes, gene expression and stabilization of redox homeostasis.

KGA is a non-toxic molecule, has good solubility and is relatively stable in aqueous solutions, and therefore might be easily applied as a dietary supplement. Inclusion of KGA in human and animal nutrition formulas is of great interest and was proved to be beneficial to health [2,3]. Studies performed on animal models showed that KGA application extended the healthspan and lifespan [4] enhanced mineralization and mechanical properties of bone tissue [5], facilitated the transport of organic anions in the kidney [6] and enhanced the metabolism of fats and prevented their peroxidative damage [7]. Human clinical studies demonstrated that KGA had a positive impact, e.g., on gut morphology and function [8], prevented the decrease in muscle protein synthesis in postoperative patients [9] and improved amino acid metabolism in patients on hemodialysis [10]. Moreover, the calcium salt of KGA prevented bone loss in osteopenic patients [11], and acted as a phosphate binder and normalized secondary hyperparathyroidism in hemodialysis patients [12].

The broad range of possible applications of KGA in medicine, pharmacy, animal and human nutrition, tissue engineering, synthesis of biopolymers, heterocycles and antibiotics [13,14,15] makes the compound particularly valuable for the industrial market. The attractiveness of the acid is apparent from the much higher price of KGA (15–20 $/kg) in comparison to citric acid (0.6 $/kg), another intermediate of the Krebs cycle [16]. However, the price level is also a consequence of difficulties in the KGA production process.

At present, KGA production is performed by multi-step chemical synthesis which uses toxic chemicals, thus generating toxic wastes, and is characterized by low selectivity of the product. KGA synthesis via biocatalysis is ecologically attractive, yet the need for special substrates and highly active enzymes hinders production on the commercial scale due to economic reasons [17,18]. Microbial fermentation techniques can use a variety of substrates, even of low purity; hence, they seem to be both an environmentally and financially friendly alternative for KGA production. However, the formation of by-products, especially other organic acids, e.g., citric and pyruvic, seems to be the greatest limitation to the attractiveness of this process [14,19].

Although various microorganisms have been reported for the ability of KGA production, the use of *Yarrowia lipolytica* yeast has drawn particular interest. The possibility of the yeast’s application for KGA biosynthesis has been studied since the 1960s [14,19]. The studies considered the use of various substrates and optimization of medium components, different cultivation techniques and metabolically engineered strains. However, to the best of our knowledge, the results obtained to date have not resulted in commercial production of KGA using *Y. lipolytica*.

The present study aimed to develop a technology for the production of dietary supplements based on yeast biomass and KGA, produced by a new transformant strain of *Y. lipolytica* with improved KGA biosynthesis ability, as well to verify the suitability of products obtained on a laboratory scale for food and feed purposes.

## 2. Results

In the first stage of the research, *Yarrowia lipolytica* Wratislavia 1.31 was used to construct strains with putative improved assimilation of fatty substrates and glycerol. The newly constructed strains overexpressed one or a combination containing two (*GUT1* and *CIT1*) and three genes (*GUT1*, *CIT1* and *YALI0E34672g*) simultaneously.

**Figure 1:**
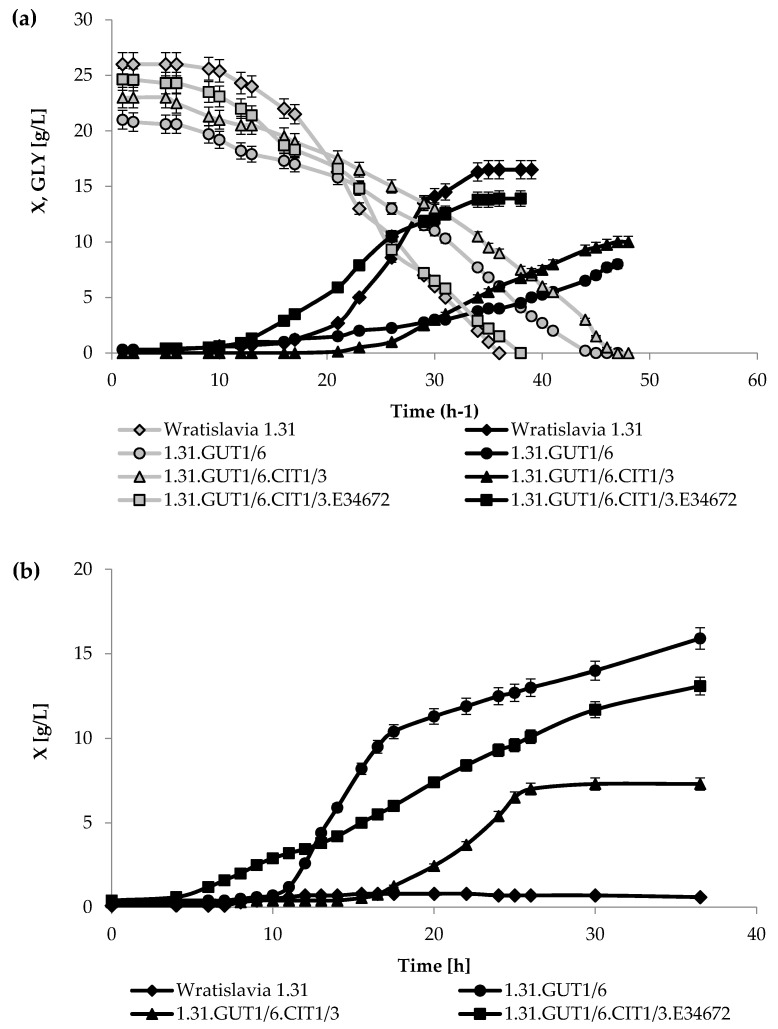
Kinetics of yeast growth (X; black lines) and glycerol (GLY; gray lines) consumption of Wratislavia 1.31 and transformant strains of *Y. lipolytica* growing on glycerol (**a**) and rapeseed oil (**b**). Error bars represent the standard deviations of duplicates.

Subsequently, *Y. lipolytica* Wratislavia 1.31 and three obtained transformant strains were compared for the ability to grow on glycerol and rapeseed oil in media containing 200 μg/L of thiamine. The growth curves are presented in Figure 1, while Table 1 compares the final biomass concentration, the maximum specific growth rate and the glycerol utilization rate for the process using this carbon source.

All strains under the applied conditions were characterized by a long lag phase, especially in the case of the 1.31.GUT1/6.CIT1/3 recombinant, which required the longest adaptation time in both media. In turn, in the case of application of both substrates, the logarithmic phase was achieved the earliest by the strain 1.31.GUT1/6.CIT1/3.E34672. Cultures in the glycerol medium were produced until the carbon source was completely depleted (Figure 1a), which was the fastest for the Wratislavia 1.31 strain, and the slowest for the 1.31.GUT1/6.CIT1/3 strain. The highest value of the maximum specific growth rate was observed in the culture of the strain 1.31.GUT1/6.CIT1/3.E34672 (Table 1). The biomass concentration in the stationary phase significantly differed among examined strains and ranged from 8.0 to 16.5 g/L for 1.31.GUT1/6 and Wratislavia 1.31 strains, respectively. The highest volumetric rate of glycerol consumption was observed in the process with Wratislavia 1.31 and the lowest for 1.31.GUT1/6.CIT1/3, while the specific rate of substrate utilization was by far the highest for the 1.31.GUT1/6 strain. When rapeseed oil was used as the carbon source (Figure 1b), cultures were produced for 37 h, and during the cultivation period the stationary phase was reached only for the strain 1.31.GUT1/6.CIT1/3. No growth was observed in this medium in the culture of Wratislavia 1.31. Interestingly, in the oil-based medium, the recombinant 1.31.GUT1/6 showed the highest concentration of biomass (15.9 g/L) and value of the maximum specific growth rate (0.54 h^−1^). When the strain 1.31.GUT1/6.CIT1/3.E34672 was cultivated on rapeseed oil medium, the process was characterized by similar growth parameters, i.e., biomass level and maximum specific growth rate, as when grown on glycerol medium (Table 1).

In order to verify whether the introduced transformations resulted in additional unexpected changes in the capability of assimilation of other carbon sources, the parental strain Wratislavia 1.31 and three obtained transformants were compared for the ability to grow on different carbon sources by performing the API 32 C test. The results demonstrated no difference in the analytical profile index among examined strains (data not shown).

**Figure 2:**
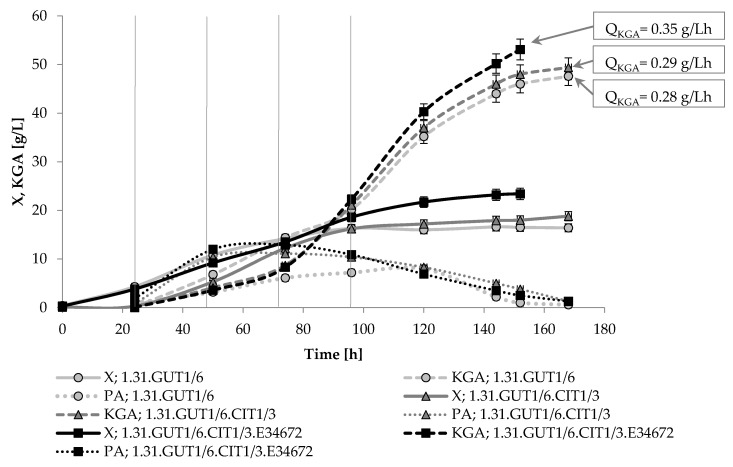
Kinetics of yeast growth (X) and production of α-ketoglutaric (KGA) and pyruvic (PA) acids by recombinant strains of *Y. lipolytica* growing on mixed (glycerol–oil) medium. Vertical lines indicate the time of dosing a portion of the substrate to the culture. Error bars represent the standard deviations of duplicates.

In the next step, the transformant strains were examined for the production of α-ketoglutarate (KGA) on media with mixed substrates (glycerol–oil) and reduced concentration of thiamine (3 μg/L). The cultivation processes were performed for 168 h and the results of yeast growth as well as KGA and pyruvic acid production kinetics are presented in Figure 2. In the conditions applied in this experiment, all the strains reached the stationary phase of growth after about 100 h of cultivation. The final biomass concentration varied among strains in the range of 16.4–23.0 g/L. and was the highest in the culture of the strain 1.31.GUT1/6.CIT1/3.E34672. This strain also demonstrated the highest KGA biosynthesis ability by producing 53.1 g/L of the acid with volumetric productivity of 0.35 g/Lh. In the case of all examined strains, pyruvic acid was initially co-produced simultaneously with KGA, but after about 60 h of the process the yeast started to consume it and the final pyruvate concentration reached not more than 0.6–1.3 g/L. In the case of cultivation of all strains, under the examined culture conditions, citric acid was not detected in the final culture broth.

**Figure 3:**
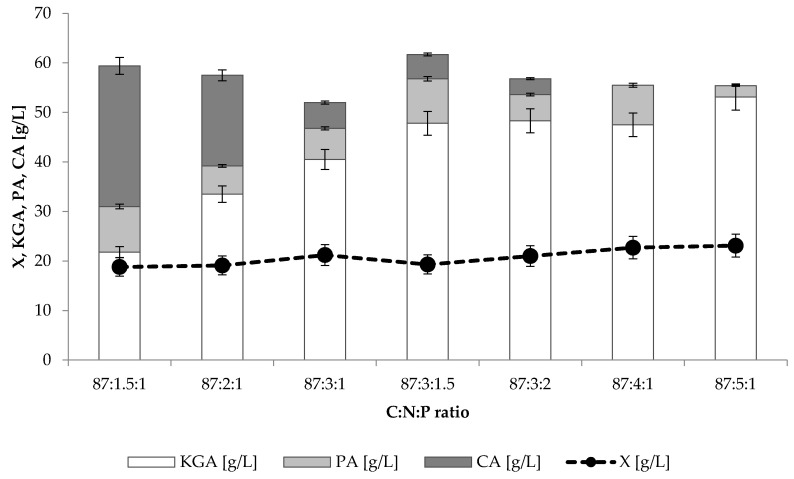
The influence of applied carbon:nitrogen:phosphorus ratio in cultivation media on yeast growth (X) and level of α-ketoglutaric (KGA), pyruvic (PA) and citric (CA) acid production by *Y. lipolytica* 1.31.GUT1/6.CIT1/3.E34672. Error bars represent the standard deviations of duplicates.

Based on the results obtained from previous experiments, the transformant strain 1.31.GUT1/6.CIT1/3.E34672 was chosen for further analysis in which the effect of different carbon:nitrogen:phosphorus (C:N:P) ratios on KGA production was studied. The results of the experiment are presented in Figure 3 and Table 2. All the cultures in this experiment were performed at the same carbon concentration (100 g/L) on mixed glycerol–oil medium (for details, see Materials and Methods) and with a thiamine concentration of 3 μg/L. Peterson and Hustrulid [20] reported that the carbon content in rapeseed oil was 79.5%. With this assumption, the amount of carbon in the used glycerol–oil medium was 39.46 g/L. With respect to this result, the changes in the C:N:P ratio of the applied media were calculated and presented in Table 2. In the cultures, the increase in nitrogen and phosphorus in the C:N:P ratio resulted only in a slight increase in biomass concentration, from 18.8 to 23.1 g/L (Figure 3). However, the protein content of biomass was observed to increase significantly, from 12.7% in the culture with a C:N:P ratio of 87:1.5:1, to 29.9% when the ratio was 87:5:1 (Table 2). The positive effect of increasing nitrogen and phosphorus in the C:N:P ratio was obvious in the case of KGA biosynthesis. The results demonstrated that within applied variants of the C:N:P ratio, the KGA content in the culture broth increased from 21.8 to 53.1 g/L. Furthermore, it was noted that the formation of by-products followed the opposite trend—both pyruvic and citric acid concentrations decreased. The enhancement of KGA biosynthesis was also shown in the volumetric productivity values, which increased from 0.18 g/Lh in the case of the 87:1.5:1 ratio, to above 0.31 g/Lh when 87:3:1.5 or higher doses of nitrogen and phosphorus were introduced to the medium. In the culture with a C:N:P ratio of 87:1.5:1, yeast produced 28.4 g/L of citric acid, whereas in the cultures with the ratios of 87:4:1 and 87:5:1, biosynthesis of this acid was not observed. Additionally, the concentration of pyruvic acid was noted to decrease, from 9.2 to 2.3 g/L, when the C:N:P ratio changed from 87:1.5:1 to 87:5:1, respectively. As a result of decreased citric and pyruvic acid content, improved selectivity of KGA biosynthesis was noted in the post-culture broth and reached the maximum level of 96%.

In the previous experiment, the protein content was observed to be the highest (29.9%) in the biomass derived from the culture of the strain 1.31.GUT1/6.CIT1/3.E34672 where the C:N:P ratio was 87:5:1. In the biomass collected after that process, the protein concentration and the amino acid profile were additionally determined (Table 3). The yeast biomass was characterized by an essential amino acid content of 44.8 g/100 g of protein, comparable to the level of the FAO/WHO whole egg standard [21]. In the analyzed biomass, the content of tryptophan and sulfur amino acids (methionine and cysteine) was significantly lower than in the FAO/WHO reference. However, the concentration of other amino acids was at a similar or much higher level when compared to the standard. The value of Oser’s essential amino acid index calculated for the analyzed biomass sample was 78.7. In order to analyze the nutritional value more deeply, the biomass was also analyzed for lipid content and fatty acid composition (Table 4). The total cellular lipid content (TCL) of the examined sample reached 20.8%. The fatty acid analysis indicated the high dominancy of oleic acid, which was accumulated in the cells at 71.6% of TCL. Additionally, significant quantities of linoleic and linolenic acid of, respectively, 9.1 and 7.0% of TCL, were detected in the analyzed biomass. It was also noted that the production of palmitic, palmitoleic and stearic acids did not exceed 2.15% of TCL.

**Figure 4:**
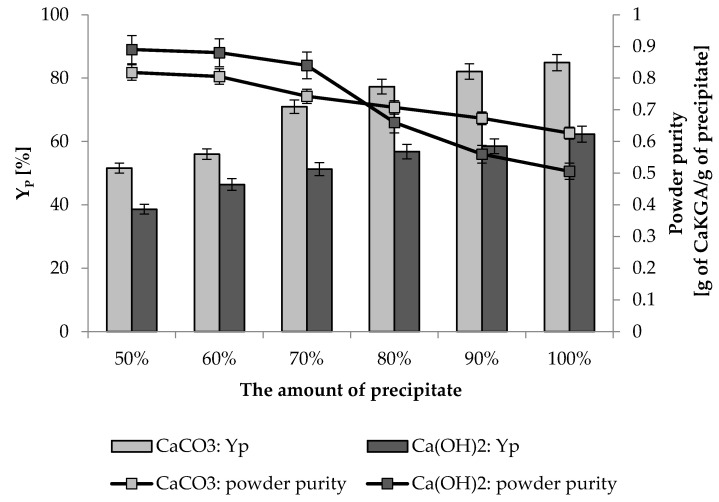
Yield of precipitation of KGA (Y_P_) and purity of CaKGA powder obtained after precipitation by CaCO_3_ or Ca(OH)_2_ used in different amounts. Error bars represent the standard deviations of duplicates.

The KGA present in the post-cultured broth was subjected to precipitation by CaCO_3_ or Ca(OH)_2_ used in the range from 50 to 100% of the amount resulting from the stoichiometry of the reaction. The results demonstrated that regardless of the agent used for precipitation, as its amount increased, the efficiency of KGA precipitation increased (Figure 4). However, higher yield of precipitation was observed in the case of CaCO_3_ than Ca(OH)_2_ application, as the parameter reached 84.9%, and 62.31%, respectively. The opposite trend was observed for the purity of the precipitated calcium α-ketoglutarate (CaKGA) powder. An increased amount of the precipitating factor resulted in decreased purity of CaKGA in the powder. The highest CaKGA purity was obtained when 50% of the precipitating agent was applied, both when using CaCO_3_ and Ca(OH)_2_. The highest purity of the CaKGA reached 89% and was obtained when the precipitation process was performed using Ca(OH)_2_.

The fixed CaKGA–biomass preparation derived by precipitation by CaCO_3_ in 60% of reaction stoichiometry from unfiltered post-culture broth was analyzed for the content of yeast biomass, CaKGA and kynurenic acid (Table 5). It was found that in the obtained preparation biomass and CaKGA content reached 22.2 and 60.5%, respectively. Moreover, in the analyzed sample, kynurenic acid was present at 87.2 μg/g of the product. In the plate test of viability, no growth was observed, indicating that the examined preparation contained only non-viable yeast cells.

## 3. Discussion

When comparing the studies concerning production of α-ketoglutarate (KGA) by *Yarrowia lipolytica* on hydrophilic and hydrophobic substrates, it can be noted that higher production parameters were obtained when the latter was applied as the carbon source [14,22]. The main problem of glycerol application for KGA biosynthesis is by-production of large quantities of pyruvic acid, which negatively affects the amount of KGA produced by the yeast and significantly reduces the selectivity of the process. However, compared to hydrophobic substrates, the possibility of using highly water-soluble glycerol as a substrate is of great practical importance, especially for industrial-scale processes. It was already demonstrated that production of KGA might be effective even when oil is partially exchanged for glycerol when both substrates are introduced simultaneously, according to the appropriate dosing strategy [23]. The subject of the present study was the use of mixed glycerol–oil-based medium for efficient KGA production. Therefore, the transformations of the yeast were aimed at improving the metabolism of both carbon sources by overexpression of genes native to *Y. lipolytica*. In order to improve the utilization of glycerol, it was decided to overexpress glycerol kinase (*GUT1*–*YALI0F00484g*), the enzyme catalyzing the phosphorylation of glycerol, which is the first step of glycerol metabolism in the *Y. lipolytica* yeast cell [24]. The wild strains of *Y. lipolytica* are known for their ability to efficiently utilize hydrophobic substrates, such as TAGs, as a result of efficient secretion of lipases (especially lipase 2—Lip2) [25]. In previous research related to the characterization of citrate synthase from *Y. lipolytica* [26], it was noted that the gene *CIT1* (*YALI0C00638g*) that encodes methylcitrate synthase, an enzyme involved in the metabolism of propionate but also having citrate synthase activity, significantly accelerates utilization of lipids by the yeast. Therefore, overexpression of the *CIT1* gene was also performed in this study, improving oil utilization for putative KGA biosynthesis. In addition to the *GUT1* and *CIT1* genes, a previously uncharacterized mitochondrial organic acid transporter (*YALI0E34672g*) was introduced to investigate whether it might affect the secretion of organic acids.

In order to examine whether the introduced genes altered the abilities of recombinant strains to assimilate the carbon sources of our interest, growth cultures were performed in media containing sole glycerol or rapeseed oil (Figure 1, Table 1). It should be noted that *Y. lipolytica* is a natural thiamine-auxotrophic yeast, and according to reports for undisturbed growth, it requires the vitamin in a concentration of 200 μg/L [27]. In the performed growth cultivations, thiamine was present in the media at a level that allows unimpeded cell growth, so objective assessment of the growth abilities of the strains was possible. The obtained transformants differed in their ability to utilize both examined carbon sources. Compared to the parental strain, very similar growth parameters, final biomass concentration and maximum specific growth rate (µ_max_) were obtained for the recombinant 1.31.GUT1/6.CIT1/3.E34672 strain when growing on glycerol. The values of the specific growth rate obtained in this study in the culture with glycerol are significantly higher than those obtained in the studies of other authors [22,28,29]. Although Wratislavia 1.31 exhibited satisfactory growth parameters in the cultures with glycerol-based medium, its further application for KGA biosynthesis in mixed media was not possible due to the lack of growth ability on oil-based medium (Figure 1, Table 1). *Y. lipolytica* Wratislavia 1.31 is an acetate-negative mutant and the observation made in this study confirmed earlier reports of the strain’s inability to utilize fatty acids [26]. Of note, the wild-type strain *Y. lipolytica* A-101, which is the parental strain of Wratislavia 1.31, was characterized by good growth on oil substrates. Interestingly, all the recombinant strains examined in this study, in contrast to their parental strain, were able to utilize the rapeseed oil. This finding is in agreement with the observation made when the impact of overexpression of the *CIT1* gene in *Y. lipolytica* Wratislavia 1.31 on isocitric acid production was examined [26]. A wider spectrum of carbon sources was analyzed using the API ID 32 C test; however, between the parental and transformant strains, no differences in the assimilation profiles were observed. In this study, it was expected that overexpression of the *CIT1* gene would improve utilization of the oil substrate and enable better growth of the transformant. Surprisingly, in the case of the 1.31.GUT1/6 strain, which overexpressed only the glycerol kinase-encoding gene, the ability of growth on oil was especially high, whereas in the case of the other two strains that additionally possess the methylcitrate synthase gene, the biomass level and specific growth rate were lower (Figure 1, Table 1). It may be possible that glycerol kinase could somehow be involved in the metabolism of TAGs, resulting in increased utilization of rapeseed oil and good yeast growth. It should be noted that the 1.31.GUT1/6 strain was further used to obtain transformants overexpressing the *CIT1* and *YALI0E34672g* genes that were integrated randomly into the genome. It is presumed that the positive effect obtained after the insertion of the *GUT1* gene was compensated by the location of the genes inserted subsequently. This finding was interesting, and attempts have already been made in our laboratories to explain the metabolic reasons for these observations.

In research on the possibility of improving the production of KGA, both random mutagenesis techniques [30] and the construction of strains with overexpression of certain genes were performed. The genetic modifications introduced in other studies were based on the nature of the substrate used, e.g., overexpression of the *PYC1* gene encoding pyruvate carboxylase [31,32], or *ACS1* encoding acetyl-CoA synthase [33], but also took into account the location of KGA formation in the cell metabolism, e.g., overexpression of genes encoding isocitrate dehydrogenase (*IDP1*) [32], citrate lyase (*ACL1*) [33], α-ketoglutarate dehydrogenase (*KGD1*, *KGD2* and *LPD1*) [34] and fumarase (*FUM1*) [31]. In thiamine-auxotrophic *Y. lipolytica* yeast, the limited thiamine concentration is the well-known key factor determining reduction in α-ketoglutarate dehydrogenase activity, thus allowing overproduction of KGA [19]. Therefore, in this study, the obtained transformants were examined for the effectiveness of KGA production on mixed glycerol–oil-based medium containing 3 μg/L of thiamine (Figure 2). The overexpression of selected genes (*GUT1*, *CIT1*, *YALI0E34672g*) resulted in obtaining strains with various abilities in KGA biosynthesis. The strain 1.31.GUT1/6 was able to produce 47.6 g/L of KGA, whereas further transformations resulted in an increase in KGA biosynthesis by 4 and 12% when a combination of two and three genes was examined, respectively. However, as explained above, under the applied conditions, comparison of transformants to the parental strain was not possible due to the lack of ability of Wratislavia 1.31 to grow on rapeseed oil. In the case of all strains, pyruvic acid formation was observed in the course of the cultivation, but before the end of the process it was almost completely utilized. Similarly, total or partial re-consumption of pyruvate was observed when glycerol was used as the substrate for KGA biosynthesis by other transformant and wild-type strains of *Y. lipolytica* [23,31,32,34]. Zeng et al. [30] reported that the application of multi-step random mutagenesis in a wild-type strain of *Y. lipolytica* WSH-Z06 made it possible to increase the production of KGA by 34%. An increase in KGA production of 33% and 35%, respectively, was noted when *ACL1* and genes encoding carboxylate transporters were overexpressed in the above mentioned wild-type parental strain [33,35]. Overexpression of *PYC1-IDP1* genes in combination in the *Y. lipolytica* H355A strain resulted in an increase in KGA production by 19% [32], while in the case of the *FUM1* gene, no increase in KGA level production was observed, although the selectivity of the process increased [31]. In turn, unfavorable changes were observed after the overexpression of the *KGD1*, *KGD2* and *LPD1* genes in *Y. lipolytica* H222-MH1, as a result of which the production of KGA decreased by 25.8%, while the pyruvate production increased 1.3-fold [34]. The achievements of *Y. lipolytica* engineering towards enhancing KGA biosynthesis are presented in Table 6.

In order to optimize the KGA production by the selected strain 1.31.GUT1/6.CIT1/3.E34672, the impact of the C:N:P ratio on the acid biosynthesis was evaluated. The usual observations in studies on the effect of the C:N relation on the biosynthesis processes with *Y. lipolytica* were reduction in growth and favored product formation when the carbon source concentration increased to a high excess over nitrogen [36,37,38,39]. This trend was not observed in this study during KGA biosynthesis, as the biomass remained at a similar level while the yeast was cultivated at an increased ratio of C:N:P (Figure 3). This might be explained by two reasons: first, in the present experiment the highest C:N ratio reached a maximum level of 58:1 (equal to the culture with C:N:P of 87:1.5:1) and was much lower than mentioned in other studies; second, in the present KGA production process the low thiamine concentration used was the factor limiting the growth of the yeast. The protein content of the biomass increased up to 29.9% with increased nitrogen availability in the culture where the C:N:P ratio reached 87:5:1 (Table 2). In processes optimized for biomass production, and thus without limiting factors and with comparatively high nitrogen availability, the protein content in the dry matter of *Y. lipolytica* has ranged from over 30% up to 54% [40,41,42,43]. The literature reports that high carbon excess stimulated different processes performed with *Y. lipolytica*, e.g., erythritol [38,39], lipid [37] and citric acid [36] production, where a C:N ratio of about 87:1, 100:1 and 367:1 was applied, respectively. In turn, overproduction of KGA requires limitation of cell growth by a low thiamine concentration combined with the maintenance of high nitrogen availability during the biosynthesis period [27]. In a previous study performed with *Y. lipolytica* A-10 under thiamine deficiency (1 μg/L), the C:N ratio, increased from 17:9 to 32:2, doubled the amount of pyruvic acid from 34.2 to 65.5 g/L and had almost no effect on the production of KGA [44]. In contrast, in the present study, increased nitrogen availability, within the examined range, generally resulted in increased production and productivity of KGA (Table 2). Moreover, the decrease in the C:N ratio resulted in improved selectivity of the process (up to 96%) as the simultaneous decrease in by-product formation was noted, especially in the case of citric acid. This observation is in accordance with previous reports where decreased nitrogen availability stimulated biosynthesis of citrate [36,39]. Nitrogen limitation, which occurred in the cultures with a lower C:N:P ratio, is a well-known factor affecting isocitrate dehydrogenase activity and membrane permeability, thus allowing citrate overproduction and secretion. As mentioned above, pyruvic acid is produced simultaneously with KGA when glycerol is used as the substrate, however, it may be re-consumed from the culture broth and metabolized to KGA (see Figure 2). In the presented experiment (Figure 3), the increased availability of nitrogen led to slight increase in yeast growth and enabled more efficient substrate consumption. Consequently, in these cultures, pyruvate was utilized faster and was present in the post-culture medium only in small amounts. A more detailed metabolic mechanism of KGA overproduction under high nitrogen concentration was described previously by Chernyavskaya et al. [27]. When biodiesel waste, containing glycerol and fatty acids, was used as a carbon source for KGA biosynthesis, within the examined range of 9:1 to 876:1, the C:N ratio of 29:1 was indicated as the most suitable [19]. In optimized medium, *Y. lipolytica* VKM Y-2412 was able to produce 80.4 g/L of KGA with productivity of 0.42 g/Lh. When the same strain was cultivated in a medium containing rapeseed oil as a sole carbon source, within 192 h of the process, 102.5 g/L of KGA was produced with a yield of 0.95 g/g [45]. In the present work, the highest parameters of KGA were obtained when the C:N:P ratio was 87:5:1 (equal to C:N ratio of 17.4:1) where 53.1 g/L of KGA corresponded to productivity of 0.35 g/Lh and a yield of 0.53 g/g. In the abovementioned reports, the overexpression of different genes enabled KGA production in the range of 44–186 g/L to be reached [31,32,33,34,35].

After biotechnological processes performed using microorganisms, especially in the case of hazardous ones, post-production biomass waste management might be problematic. It is of great importance that the processes carried out using non-pathogenic *Y. lipolytica* were granted “generally recognized as safe” (GRAS) status by the US Food and Drug Administration. Moreover, the safety of application of the yeast in food and feed was proved as well [46]. In 2010, the biomass of *Y. lipolytica* was approved for use as feedstuff by the European Feed Manufacturers’ Federation (00575-EN). Recently, the biomass of *Y. lipolytica* has been authorized on the market as a novel food intended for human nutrition by the Commission Implementing Regulation (EU) 2019/760. According to EU Regulation (EC) No 1829/2003, the application of genetically engineered biomass in food and feed is possible after authorization by the European Food Safety Authority. Nevertheless, the usefulness of biomass for consumption purposes depends on its nutritional value. In the present study, the best KGA biosynthesis parameters and the highest protein content were observed in the culture with a C:N:P ratio of 87:5:1. Therefore, in the biomass collected after the described process, the nutritional value was assessed by more detailed protein and lipid analysis. The content of essential amino acids (Table 3), which determines the quality of protein, reached 47.0 g/100 g of protein and was very similar to the value of FAO/WHO whole egg standard (51.2 g/100 g of protein) and almost four times higher than the standard required for mature humans (12.7 g/100 g of protein) [21]. The essential amino acid index, calculated according to Oser’s method, reached 80.8 and 307.4 when a whole egg and adult requirement standard was used, respectively. The level of this parameter for *Y. lipolytica* grown on glycerol was reported as 67.2–72.3 [41,42]. Compared to the whole egg reference, in the examined biomass, higher contents of isoleucine, threonine and lysine of, respectively, 7.45, 6.23 and 7.84 g/100 g of protein, were recorded. In the biomass, the limiting amino acids were methionine and cysteine, the pool of which reached 1.6 g/100 g of protein, corresponding to a chemical score of 28.1 and 94.1 calculated with respect to the FAO/WHO standards for whole egg and adult men’s requirements, respectively. The low sulfuric content of the *Y. lipolytica* yeast was also noted in previous reports [40,41,42]. For animal feed, the recommended level of sulfur amino acids is 3.5 g/100 g of protein [47]. The results obtained in this study did not meet this criterion for the biomass to be used as feed alone. However, taking into account the composition of exogenous amino acids reported for cereals, it can be concluded that *Y. lipolytica* biomass is an ideal component of cereal-based feeds, which are characterized by a high content of sulfur amino acids, but due to the low content of isoleucine, lysine and threonine, require their supplementation [48].

In the examined biomass, the total lipid content, the other factor affecting quality of biomass, reached 20.8% in the dry biomass weight (Table 4), and was comparable to or even higher than the results presented for *Y. lipolytica* by other researchers, where the lipid content was in the range 6.5–20.3% [40,41,42,49]. It is worth noting that in the present study, the yeast was cultivated in conditions of high nitrogen availability, whereas lipid accumulation is favored when nitrogen is limited in the culture medium. According to the literature reports, under nitrogen deficiency and in optimized conditions, *Y. lipolytica* may accumulate lipids up to 47.5% when cultivated on glycerol [50] or even up to 60% in the case of oil-based media application [51,52]. In this work, the analysis of fatty acid profile showed a very high predominance of oleic acid (71.6%) in the total cellular lipids (Table 4). Significant amounts of linoleic and linolenic acids were also noted. Summing up, almost 90% of the lipids present in the biomass were unsaturated acids, which is a great advantage when application in human or animal nutrition is considered. Large amounts of oleic and linoleic acids were also observed in previous studies [40,41,50]. Moreover, the same trend of fatty acid profile in lipids was observed by Michalik et al. [42], who studied and advised the use of biomass of *Y. lipolytica* for animal feeding. The genetically modified *Y. lipolytica* biomass, rich in eicosapentaenoic acid, was applied and demonstrated its value as an efficient ω-3 feed supplement for salmon aquaculture [53,54] and has been marketed by AquaChile (Puerto Montt, Chile) since 2010 [55].

In 2012, the method for preparing calcium α-ketoglutarate (CaKGA) was described as a complicated, multistep process using hazardous substances, i.e., sodium methoxide, methyl dichloroacetate or methyl acrylate [56]. Recently, a two-step method was proposed in which KGA reacts with the metal salt of an acid (lithium, sodium or potassium salts of carbonate or bicarbonate) and then contacts the calcium salt of acetate, formate or chloride [57]. In this study, a simple method of KGA precipitation by reacting with Ca(OH)_2_ or CaCO_3_ was proposed. In terms of purity, CaKGA products obtained by both methods were comparable but the use of CaCO_3_ allowed for higher process efficiency (Figure 4). It should be mentioned that when CaCO_3_ was applied in the process, the purity of the precipitate reached about 80% and the unreacted portion of the precipitation reagent was present as an impurity in the product. It is worth noting that calcium carbonate is a safe and regular feed component. In feeding, it is commonly introduced in the form of fodder chalk added to balance animals’ diets with calcium and prevent metabolic disorders, resulting in poor feed utilization and bone loss, especially in young livestock [58,59]. Calcium carbonate is also applied in medicine, e.g., for prevention or therapy of osteoporosis [60].

The possibility of using biomass as a component of a dietary supplement makes it possible to prepare the CaKGA preparation containing yeast cells, directly from the post-culture fluid. Avoiding the energy-consuming and problematic biomass filtration process, especially in industrial conditions, is economically advantageous and renders the process competitive. It is important that the yeast biomass obtained in this study was characterized by good parameters of content and composition of protein and lipid; therefore, its presence in the product increases its nutritional value. However, it should be noted that according to the European Food Safety Authority Panel on Nutrition [61], the application of *Y. lipolytica* biomass as a novel food requires the absence of viable cells. Therefore, the fixed CaKGA–biomass preparation was checked for viability of the yeast cells, and the results demonstrated that it meets the stated criterion (Table 5). Another advantage of the obtained preparation is the presence of kynurenic acid. The health-promoting effect of this bioactive compound results, among other factors, from its antioxidative, neuroprotective, hepatoprotective, anticancerogenic, anti-inflammatory, analgesic, anticonvulsant, anti-ulcerative and antiatherogenic properties [62]. Although valuable properties of this acid have been demonstrated, the possibility of supplementing it with a daily diet is limited by the low content in natural products. It was reported that among the easily available daily food products examined, the amount of kynurenic acid ranged from 0.006 nmol/g in red paprika to 2.2 nmol/g in broccoli [63]. Unprecedented in other food products, the highest reported kynurenic acid content of 601 μg/g was observed in Italian chestnut honey [64]. *Y. lipolytica* yeast has already been proved as a suitable platform for kynurenic acid production, as in optimized conditions, with the use of tryptophan-supplemented media, the kynurenic acid content in biomass reached 494.16 μg/g [65]. In this study, in the fixed CaKGA–biomass preparation, 87.2 μg/g of kynurenic acid was obtained (Table 5), which makes it a valuable source of this bioactive acid and represents an additional health benefit of the described product.

## 4. Materials and Methods

### 4.1. Microorganism

The acetate-negative mutant strain *Yarrowia lipolytica* Wratislavia 1.31 and three recombinant strains of *Y. lipolytica*—1.31.GUT1/6, 1.31.GUT1/6.CIT1/3, 1.31.GUT1/6.CIT1/3.E34672 — were used in this study. The strains belong to the culture collection of the Department of Biotechnology and Food Microbiology at the University of Environmental and Life Sciences (Wrocław, Poland) and were stored on YM agar slants at 4 °C. Additionally, the strain 1.31.GUT1/6.CIT1/3.E34672 was deposited in the Westerdijk Fungal Biodiversity Institute (registered as *Y. lipolytica* CBS146773). The genotype characteristics of the strains used in the study are presented in Table 7.

### 4.2. General Genetic Techniques and Plasmid Construction

Standard molecular genetic techniques were used throughout this study following Sambrook and Russell [66]. Restriction enzymes were purchased from New England Biolabs (Ipswich, England). Genomic DNA from *Y. lipolytica* Wratislavia 1.31 was prepared as described by Querol et al. [67]. PCR amplification was performed using a T-Personal thermal cycler (Biometra, Analytik Jena GmbH, Germany) and Q5 (New England BioLabs, MA, USA) DNA polymerases. PCR fragments were then purified with a Qiagen Purification Kit (Qiagen, Hilden, Germany), and DNA fragments were recovered from agarose gels using a QIAquick Gel Extraction Kit (Qiagen, Hilden, Germany). The Staden software package (MRC, Cambridge, England) was used for gene sequence analysis [68]. Three genes were analyzed: glycerol kinase (*GUT1*, *YALI0F00484g*), methylcitrate synthase (*CIT1*, *YALI0E00638g*) and non-characterized mitochondrial transporter (*YALI0E34672g*). To amplify the genes from genomic DNA of the *Y. lipolytica* Wratislavia 1.31 strain, the primers listed in Table 8 were used. The genes were digested with the corresponding restriction enzymes (Table 8) and cloned into the JME1128 plasmid [69,70], that had been digested with *BamHI-AvrII* restriction enzymes. Only URA3ex plasmids, containing an excisable *URA3* expression cassette, were used during this study. To delete the *URA3* gene (*YALI0E26741g*) and obtain an auxotrophic strain from *Y. lipolytica* Wratislavia 1.31, the disruption cassettes were prepared in accordance with the protocol of Fickers et al. [71] using the invertase coding gene (*SUC2*) from *Saccharomyces cerevisiae*, allowing for growth on sucrose as the sole carbon source. The *SUC2* expression cassette was further bordered with LoxP sequences recognized by Cre recombinase, allowing its excision.

**Figure 5:**
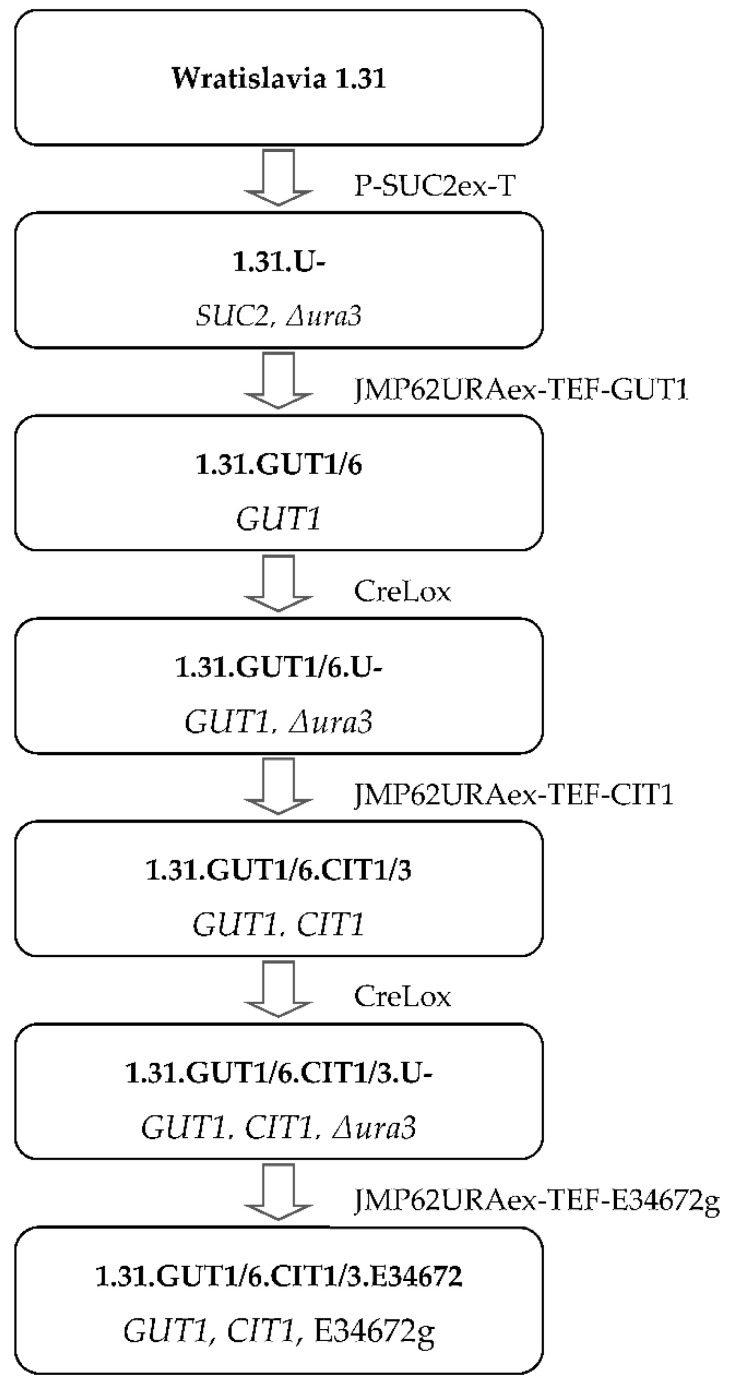
The schematic representation of obtaining transformants of *Y. lipolytica* Wratislavia 1.31.

The URA3-negative transformant of *Y. lipolytica* Wratislavia 1.31 was created using the P-SUC2ex-T cassette and selected on a minimal YNB medium with sucrose (2%) [26]. This strain was further used to obtain transformants overexpressing a combination of all three genes (*GUT1*, *CIT1* and *YALI0E34672g*) under the control of the constitutive TEF promoter. Transformation of *Y. lipolytica* was performed with the lithium acetate procedure [72], using NotI-digested fragments for random chromosomal integration [73]. All transformants were PCR verified. Auxotrophies were restored via *URA3* excision using the Cre-lox recombinase system following transformation with the replicative plasmid pUB4-Cre1 (JME547) [71]. The scheme for obtaining transformant strains is depicted in Figure 5.

### 4.3. Media and Culture Conditions

Inoculation cultures were performed in 300-mL Erlenmeyer flasks containing 50 mL of a medium consisting of (g/L): edible rapeseed oil or pure glycerol (98%; Wratislavia-Bio; Wrocław, Poland)—20.0; NH_4_Cl—7.0; KH_2_PO_4_—2.0; MgSO_4_·7H_2_O—1.2; Ca(NO_3_)_2_—0.8; CaCO_3_—10.0; and thiamine—200 or 3 μg/L (when used as inoculum for growth or α-ketoglutarate (KGA) production cultures, respectively) dissolved in distilled water. The cultures were grown for 72 h on a rotary shaker (CERTOMAT IS, Sartorius, Germany) at 29.5 °C and 140 rpm.

The growth ability of the parental and recombinant strains was compared in batch cultures in a 5-L bioreactor (BIOSTAT B Plus, Sartorius, Germany) containing 1.75 L of the following medium (g/L): edible rapeseed oil or pure glycerol—20.0; NH_4_Cl—7.0; KH_2_PO_4_—2.0; MgSO_4_·7H_2_O—1.2; Ca(NO_3_)_2_—0.8; and thiamine—200 μg/L, dissolved in tap water. The bioreactor culture was started by introduction of 150 mL of the shake-flask inoculation culture. In the bioreactor process, the following culture conditions were applied: temperature of 29 °C, agitation rate of 800 rpm, aeration rate of 0.8 vvm and pH 4.5 maintained by the addition of 15% KOH. Samples from bioreactor cultures were taken with the frequency indicated in the figures in the “Results” section.

KGA production ability of recombinant strains and the effect of the carbon to nitrogen to phosphorus ratio (C:N:P) on KGA production by *Y. lipolytica* 1.31.GUT1/6.CIT1/3.E34672 were studied in the same fermenter as mentioned above containing 2 L of the bioreactor production medium with glycerol (20.0 g/L) and a thiamine concentration of 3 μg/L. In these experiments, the culture was additionally fed by 4 portions of 20 g/L of mixed substrates (glycerol—10 g/L and rapeseed oil—10 g/L) at 24, 48, 72 and 96 h of cultivation. The cultivation parameters were the same as described above, except the pH, which was maintained at 3.5 throughout the process by addition of 15% Ca(OH)_2_. The details on preparing different C:N:P ratios in the experiments are provided in the “Results” section.

In the CaKGA–biomass precipitate, the assessment of yeast viability was performed using YPG medium with the following composition: glucose—20 g/L, yeast extract—3 g/L, bactopeptone—3 g/L and agar—20 g/L dissolved in distilled water. A precipitate sample of 1 g was suspended in 100 mL of sterile distilled water, mixed and 0.2 mL was used to inoculate the plate medium. After 48 h of incubation in a laboratory thermostat (Memmert GmbH, Schwabach, Germany) at 30 °C, the number of viable microorganisms was determined by determining the colony-forming units per gram of the analyzed sample (cfu/g). All chemicals used in the study were of analytical purity (Sigma-Aldrich, Sternheim, Germany).

### 4.4. Comparison of Carbon Source Assimilation

The ability of assimilation of different carbon sources was examined using the API ID 32C fungal identification system, performed according to the procedure provided by the manufacturer (bioMérieux, Marcy l’Etoile, France).

### 4.5. Precipitation of CaKGA

The post-culture broth containing KGA was collected, yeast cells were removed by membrane filtration (pore size of Ø = 0.22 μm; Merck, Darmstadt, Germany) and the obtained fluid was analyzed for KGA concentration by the HPLC method [23]. Next, the fluid was divided into 200 mL portions to which various amounts (50–100%) of pure CaCO_3_ or Ca(OH)_2_ were added. The amount of precipitation agent was calculated taking into account the stoichiometry of the reaction and the amount of KGA in the fluid. The theoretical amount necessary for the complete precipitation of the KGA contained in the fluid was taken as 100%. After adding the precipitation factor, the suspensions were mixed for 1 h on a magnetic stirrer (500 rpm) and left for a further 24 h at 22 °C. The samples were then filtered using filter paper, washed twice with distilled water and the precipitate was collected, air-dried for 96 h and then dried to a constant weight at 105 °C. To determine the precipitate purity, 1.0 g of the powder sample was dissolved in 2 mL of HCl solution (1:1) and distilled water, in a final volume of 25 mL. The solution prepared in this way was analyzed for KGA concentration and calcium α-ketoglutarate(CaKGA) content was calculated for 1 g of analyzed powder. The yield of precipitation was expressed as the percentage ratio of the amount of KGA in the precipitate to the KGA content in the fluid before precipitation.

### 4.6. Fixed Preparation Containing Yeast Biomass and CaKGA

In order to obtain the CaKGA precipitate containing the yeast biomass, a volume of 1 L of post-culture broth was used and processed according to the precipitation method described above, omitting the biomass filtration step. In this experiment, CaCO_3_ was used in the amount of 60% of the reaction stoichiometry. After precipitation, the yeast biomass together with the water-insoluble CaKGA salt was separated on filter paper, transferred to a cuvette in which it was dried at 55 °C for 48 h and finally pulverized in a laboratory mill (ZBPP, Bydgoszcz, Poland).

### 4.7. Analytical Methods

Preparation of the samples collected from bioreactor cultures and their further analysis for biomass dry weight, glycerol, KGA and by-product (pyruvic and citric acids) concentrations was performed according to methods described previously [23]. Protein concentration and amino acid profile were analyzed and calculated according to Juszczyk et al. [41], whereas lipid concentration and fatty acid profile as well as analysis of kynurenic acid presence were determined using methodology described by Wróbel-Kwiatkowska et al. [65]. Data are presented as mean values ± standard deviation of two independent replicates.

## 5. Conclusions

In this study, the use of new engineered strains of *Y. lipolytica* with overexpression of three genes encoding glycerol kinase (*GUT1*), methylcitrate synthase (*CIT1*) and mitochondrial organic acid transporter (*YALI0E34672g*) was proposed for α-ketoglutarate (KGA) biosynthesis on a medium with mixed renewable carbon sources (60 g of glycerol + 40 g of rapeseed oil). All transformants were able to utilize both used substrates and to synthesize KGA, but they differed in growth and biosynthesis parameters. The best KGA production was demonstrated by *Y. lipolytica* 1.31.GUT1/6.CIT1/3.E34672, which overexpressed all three genes. Application of this strain allowed production of up to 53.1 g/L of KGA from 100 g/L of mixed substrates. Despite the satisfactory results, further studies are being conducted in our laboratories to optimize the conditions of KGA biosynthesis, which are necessary to fully exploit the metabolic potential of the transformants. In the present study, the possibility of KGA precipitation from the culture broth, in the form of calcium salt, by a simple method using CaCO_3_, was also demonstrated. The biomass collected after KGA biosynthesis contained 29.9% protein and 20.8% lipids, a very good profile of amino acids and high content of unsaturated fatty acids, which support its application in human and animal nutrition.

In summary, the described technology allowed us to obtain three different products with high nutritional and health-promoting properties that might be of significant interest for creating dietary supplements: first, calcium α-ketoglutarate (CaKGA) with purity of 89.9%; second, yeast biomass with good nutritional value, which might be used for food and feed applications; and third, obtained by direct simultaneous precipitation of KGA and yeast biomass from culture broth, a fixed CaKGA–biomass preparation in which the presence of kynurenic acid (87.2 μg/g) was proved, which provides additional health benefits with consumption of this product.

## Figures and Tables

**Table 1:** Comparison of growth kinetic parameters of Wratislavia 1.31 and transformant strains of *Y. lipolytica* growing on glycerol and rapeseed oil.

Carbon Source	Glycerol				Rapeseed Oil	
Parameter/Strain	X [g/L]	µ_max_ [h^−1^]	Q_GLY_ [g/Lh]	q_GLY_ [g/gh]	X [g/L]	µ_max_ [h^−1^]
Wratislavia 1.31	16.5 ± 1.4	0.25	0.74	0.044	No growth	
1.31.GUT1/6	8.0 ± 0.6	0.26	0.57	0.071	15.9 ± 1.1	0.54
1.31.GUT1/6.CIT1/3	10.0 ± 0.8	0.22	0.50	0.050	7.3 ± 0.4	0.25
1.31.GUT1/6.CIT1/3.E34672	13.9 ± 0.7	0.28	0.66	0.047	13.1 ± 1.0	0.28

X—biomass; µ_max_—maximum specific growth rate; Q_GLY_—volumetric consumption rate of glycerol; q_GLY_—specific consumption rate of glycerol.

**Table 2:** Comparison of parameters obtained after α-ketoglutaric acid production by by *Y. lipolytica* 1.31.GUT1/6.CIT1/3.E34672 in media with different carbon:nitrogen:phosphorus ratios.

NH_4_Cl [g/L]	KH_2_PO_4_ [g/L]	C:N:P Ratio	Q_KGA_ [g/Lh]	S_KGA_ [%]	TPC [%]
2.6	2.0	87:1.5:1	0.18	37	12.7 ± 0.85
3.5	2.0	87:2:1	0.28	58	20.3 ± 1.84
5.2	2.0	87:3:1	0.34	78	24.1 ± 0.57
5.2	3.0	87:3:1.5	0.33	79	21.7 ± 2.69
5.2	4.0	87:3:2	0.34	85	25.1 ± 1.70
7.0	2.0	87:4:1	0.33	80	26.5 ± 2.40
9.0	2.0	87:5:1	0.35	96	29.9 ± 1.12

Q_KGA_—KGA volumetric production rate; S_KGA_—selectivity of KGA (expressed as % of KGA in the pool of products); TPC—total protein content (expressed as % of protein in dry biomass weight).

**Table 3:** Comparison of protein content and essential amino acid profile of *Y. lipolytica* 1.31.GUT1/6.CIT1/3.E34672 after cultivation on mixed (glycerol–oil) medium.

	*Y. lipolytica* 1.31.GUT1/6.CIT1/3.E34672	Whole Egg ^1^	Adult Requirement ^1^
**TPC [%]**	29.9 ± 1.12		
**Amino acid [g/100 g of protein]**			
Histidine	2.19 ± 0.13	2.2	1.6
Isoleucine	7.45 ± 0.44	5.4	1.3
Leucine	8.10 ± 0.43	8.6	1.9
Lysine	7.84 ± 0.29	7.0	1.6
Methionine/Cysteine	1.60 ± 0.03	5.7	1.7
Phenylalanine/Tyrosine	7.00 ± 0.44	9.3	1.9
Threonine	6.23 ± 0.21	4.7	0.9
Tryptophan	0.69 ± 0.05	1.7	0.5
Valine	5.86 ± 0.27	6.6	1.3
**Nutritional values**			
*ΣEAA*	47.0	51.2	12.7
*CS* (Met + Cys)	28.1 ^2^/94.1 ^3^		
*EAAI*	80.8 ^2^/307.4 ^3^		

TPC—total protein content; *ΣEAA*—sum of essential amino acids; *CS*—chemical score (CS = (a_i_/a_s_) × 100; a_i_—concentration ratio of the restrictive amino acid; a_s_—concentration of this amino acid in the standard; *EAAI*—Oser’s essential amino acid index. ^1^ FAO/WHO standard [21]. Standards used in calculations: ^2^ whole egg; ^3^ adult requirement.

**Table 4:** Composition of fatty acids of *Y. lipolytica* 1.31.GUT1/6.CIT1/3.E34672 biomass after cultivation on mixed (glycerol–oil) medium.

TCL [%]	20.8 ± 0.49
**Fatty acids [% of TCL]**	
16:0	2.15 ± 0.32
16:1	0.99 ± 0.08
18:0	1.39 ± 0.08
18:1	71.6 ± 0.08
18:2	9.08 ± 0.08
18:3	6.97 ± 0.08
*SFA*	3.54
*MUFA*	72.59
*PUFA*	16.05

TCL—total cellular lipids (expressed as % of lipid in dry biomass weight); *SFA*—saturated fatty acidc; *MUFA*—monounsaturated fatty acids, *PUFA*—polyunsaturated fatty acids.

**Table 5:** Characteristics of the fixed CaKGA–biomass preparation obtained from the culture of *Y. lipolytica* 1.31.GUT1/6.CIT1/3.E34672.

Fixed CaKGA–Biomass Preparation	
CaKGA [%]	60.5 ± 2.4
Yeast biomass content [%]	22.2 ± 1.9
Viable cell content [cfu/1 g of the product]	not detected
Kynurenic acid [μg/g]	87.2 ± 4.3

**Table 6:** The summary of prior studies on engineering *Y. lipolytica* yeast for enhancing the biosynthesis of KGA.

Strain	Overexpressed Gene	Carbon Source	KGA [g/L]	PA [g/L]	Q_KGA_ [g/Lh]	Y_KGA_ [g/g]	Reference
H355	parental strain	R-GLY	133.0	1.9	1.51	0.47	[31]
H355A(FUM1) T1	*FUM1*		134.1	0.4	1.51	0.47	
H355A(PYC1) T3	*PYC1*		126.9	2.3	1.31	0.42	
H355A(FUM1-PYC1) T4	*FUM1*-*PYC1*		138.0	2.3	1.51	0.52	
H355	parental strain	R-GLY	156.9	8.0	1.47	0.30	[32]
H355A(IDP1) T1	*IDP1*		167.6	8.0	1.58	0.35	
H355A(PYC1-IDP1) T5	*PYC1*-*IDP1*		186.0	8.0	1.75	0.36	
*Y. lipolytica*-CON	parental strain	GLY	42.4	35.1	0.29	0.42	[33]
*Y. lipolytica*-ACS1	*ACS1*		52.6	25.4	0.37	0.53	
*Y. lipolytica*-ACL	*ACL*		56.5	20.2	0.39	0.57	
H222	parental strain	GLY	97.0	52.0	0.90	n.s.	[34]
H222-MH1	*KGD1*-*KGD2*-*LPD1*		72.0	66.0	0.70	n.s.	
WSH-Z06	parental strain	GLY	36.6	17.8	n.s.	n.s.	[35]
T1	*YALI0B19470g* (carboxylate transporter)		46.7	12.3	n.s.	n.s.	
T5	*YALI0D20108g* (carboxylate transporter)		44.0	23.5	n.s.	n.s.	
1.31.GUT1/6.CIT1/3.E34672	*GUT1*-*CIT1*-*YALI0E34672g*	GLY + O	53.1	2.3	0.35	0.53	this study

R-GLY—raw glycerol; GLY—glycerol; O—rapeseed oil; Q_KGA_—KGA volumetric production rate; Y_KGA_—yield of KGA production; n.s.—not specified.

**Table 7:** The genotype characteristics of *Y. lipolytica* strains used in this study.

Strain	Genotype
Wratislavia 1.31	An acetate-negative mutant, uracil prototroph
1.31.U-	*Δura3*, TEF-*SUC2*
1.31.GUT1/6	TEF-*GUT1*
1.31.GUT1/6.CIT1/3	TEF-*GUT1*, TEF-*CIT1*
1.31.GUT1/6.CIT1/3.E34672	TEF-*GUT1*, TEF-*CIT1*, TEF-*E34672g*

**Table 8:** Restriction enzymes used for plasmid preparation.

Primer	Restriction Enzyme Used	Sequence
GUT1-F	BclI	GAGATGATCAATGTCTTCCTACGTAGGAGCTCTCG
GUT1-R	AvrII	GAGTCCTAGGTTACTCAAGCCAGCCAACAGCTC
CIT1-F	BclI	CGCGTGATCAATGATCCCTCTTCGAACC
CIT1-R	AvrII	GCGCCCTAGGTTATTTGGCGACCTTAATAATCTC
E34672-F	BamHI	GAGAGGATCCATGGCTGCTGACGGAAAGAAG
E34672-R	AvrII	GAGGCCTAGGTTACTCCTCAAACTGGGCAGCAAAAG
